# Artificial intelligence−powered electrochemical sensor: Recent advances, challenges, and prospects

**DOI:** 10.1016/j.heliyon.2024.e37964

**Published:** 2024-09-14

**Authors:** Siti Nur Ashakirin Binti Mohd Nashruddin, Faridah Hani Mohamed Salleh, Rozan Mohamad Yunus, Halimah Badioze Zaman

**Affiliations:** a Institute of Informatics and Computing in Energy (IICE), Department of Computing, College of Computing & Informatics, Universiti Tenaga Nasional, 43000, Kajang, Selangor Darul Ehsan, Malaysia; b Fuel Cell Institute, Universiti Kebangsaan Malaysia, 43600, Bangi, Selangor, Malaysia

**Keywords:** Electrochemical, Artificial neural networks, Genetic algorithm, Energy saving, Machine learning

## Abstract

Integrating artificial intelligence (AI) with electrochemical biosensors is revolutionizing medical treatments by enhancing patient data collection and enabling the development of advanced wearable sensors for health, fitness, and environmental monitoring. Electrochemical biosensors, which detect biomarkers through electrochemical processes, are significantly more effective. The integration of artificial intelligence is adept at identifying, categorizing, characterizing, and projecting intricate data patterns. As the Internet of Things (IoT), big data, and big health technologies move from theory to practice, AI-powered biosensors offer significant opportunities for real-time disease detection and personalized healthcare. Still, they also pose challenges such as data privacy, sensor stability, and algorithmic bias. This paper highlights the critical advances in material innovation, biorecognition elements, signal transduction, data processing, and intelligent decision systems necessary for developing next-generation wearable and implantable devices. Despite existing limitations, the integration of AI into biosensor systems shows immense promise for creating future medical devices that can provide early detection and improved patient outcomes, marking a transformative step forward in healthcare technology.

## 1 Introduction

Sensors are appropriate data collection devices for the implementation of smart cities due to their distinctive place at the connection between the analogue world we stay in and the fundamental communication infrastructures [1,2]. Sensors are classified into physical and virtual sensors based on application scenarios [3,4]. One of the most significant areas is biosensors, which have evolved from conventional electrochemical biosensors to implantable and wearable biosensors. They are mostly used in healthcare [5,6], disease diagnosis [7], food security, environmental monitoring [8], and biosafety [9].

The development of electrochemical biosensors for a range of uses, including environmental monitoring and medical diagnostics, has drawn increasing attention in recent years. Recent advances in electrochemical biosensing, such as electrode substrate fabrication, miniaturization, materials science, digitalization, and artificial intelligence, have transformed our understanding of the field [10]. These biosensors identify and measure certain analytes in a sample by combining biological recognition components with electrochemical methods [11]. The application of artificial intelligence to improve the functionality and performance of electrochemical biosensors is one area of advancement in this discipline [12]. Researchers hope to increase electrochemical biosensors' sensitivity, selectivity, and overall accuracy by incorporating artificial intelligence into them [13].

The detection and monitoring of hydrogen energy is one particular application where the integration of artificial intelligence has demonstrated potential [14]. Improvement of the performance and accuracy of electrochemical biosensors by altering the nickel electrode used for electrochemical hydrogen energy detection. The redesigned nickel electrode detects hydrogen energy with greater sensitivity and selectivity, allowing for more precise measurements [15]. Furthermore, the incorporation of artificial intelligence into electrochemical biosensors enables data processing and pattern identification, resulting in real-time monitoring and prediction capabilities. This combination of modern technology and electrochemical biosensors has the potential to transform the field of hydrogen energy detection by providing a more precise and efficient means of monitoring hydrogen energy levels [16].

Artificial intelligence (AI)-powered electrochemical biosensors improve sensing performance [13,17]. AI algorithms have greatly enhanced the sensitivity and selectivity of electrochemical biosensors [18,19]. Artificial intelligence may extract useful information from complex electrochemical signals using modern data processing techniques, resulting in increased detection limits and accuracy. Thus, one of the major advancements is the integration of artificial intelligence with electrochemical biosensors for real-time monitoring of biological analytes. AI algorithms can continually examine data streams from biosensors, allowing for rapid detection of changes in analyte concentrations. This functionality is particularly useful for medical diagnostics and environmental monitoring. Advances in microfabrication techniques and AI-driven signal processing have made it easier to construct miniature electrochemical biosensors and wearable devices. These miniature and portable systems provide biomarker monitoring on-the-go, making them ideal for point-of-care diagnostics and personalized health monitoring. Next, multimodal sensing has been allowed by the integration of AI with electrochemical biosensors, allowing for the detection of numerous analytes at once. This multiplexing feature improves the efficiency and versatility of biosensing devices, enabling the complete investigation of complicated biological samples. Adaptive learning with AI-powered electrochemical biosensors can adjust to changing environmental circumstances and sample matrices. Machine learning algorithms may constantly learn from fresh data, increasing the resilience and reliability of biosensor readings over time [20].

The challenges encountered with AI-powered electrochemical biosensors were data interpretation, integration, power consumption, user acceptance, regulatory compliance, and cost-effectiveness [21]. The standardization of data collection and processing processes is critical for repeatability and cross-platform comparisons. Sustainable sensing relies on balancing computational demand with energy limits. User acceptability and regulatory compliance are critical to trust and confidence. Cost-effective technologies and scalable manufacturing procedures are required to make AI-powered biosensors available to a larger population and enable large-scale deployment [[20], [21], [22]].

**Fig. 1:**
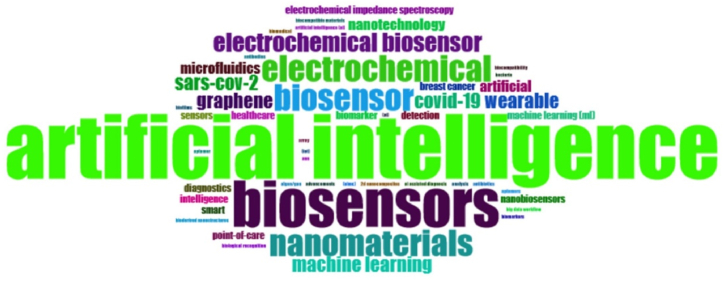
A schematic illustration of the word cloud serves as the outline in this review for the field of electrochemicals using AI.

Here, we did a series of focused keyword searches that included terms like “biosensor”. To narrow our focus on electrochemistry and machine learning, we added terminology like “artificial intelligence” and “artificial neural network” that are commonly used in the electrochemical field. As shown in Fig. 1, this word cloud was created by examining the primary references used in this study. The word cloud depicts the most commonly occurring words, with each word's size proportionate to its frequency of appearance. Larger terms in the cloud imply a greater prevalence in this review, providing an overview of the key themes and concepts driving our research.

The objective of this paper is to provide an overview of artificial intelligence (AI)-based electrochemical techniques for disease forecasting and evaluation. A diverse group of biosensor and AI writers contributed ideas and thoughts for the AI-based analytical approaches discussed in this article. Section 2 provides a brief overview of artificial intelligence in the electrochemical sector. Section 3 explains the various cutting-edge approaches utilized to examine the combination of AI and electrochemical techniques. Section 4 summarizes the use of electrochemical sensors in the detection of disease-related biomarkers. In Section 5, we explain how electrochemical sensors and AI can help clinicians make decisions about acceptable treatment alternatives. Finally, we discuss the findings from the study challenges, together with recommendations for future research areas.

## 2 Electrochemical biosensors

### 2.1 Introduction to electrochemical biosensors

Electrochemical biosensors are analytical tools that identify and measure target analytes by combining components of biological recognition with electrochemistry [23]. A working electrode, a reference electrode, and a counter electrode make up each of them. They are employed in drug research, environmental pollutants [24], food safety [25], and medical diagnostics because of their excellent sensitivity, specificity, and quick reaction times. Data interpretation, standardization, integration, power consumption, cost-effectiveness, and regulatory compliance are among the difficulties.

### 2.2 Types and key components of electrochemical biosensors

**Fig. 2:**
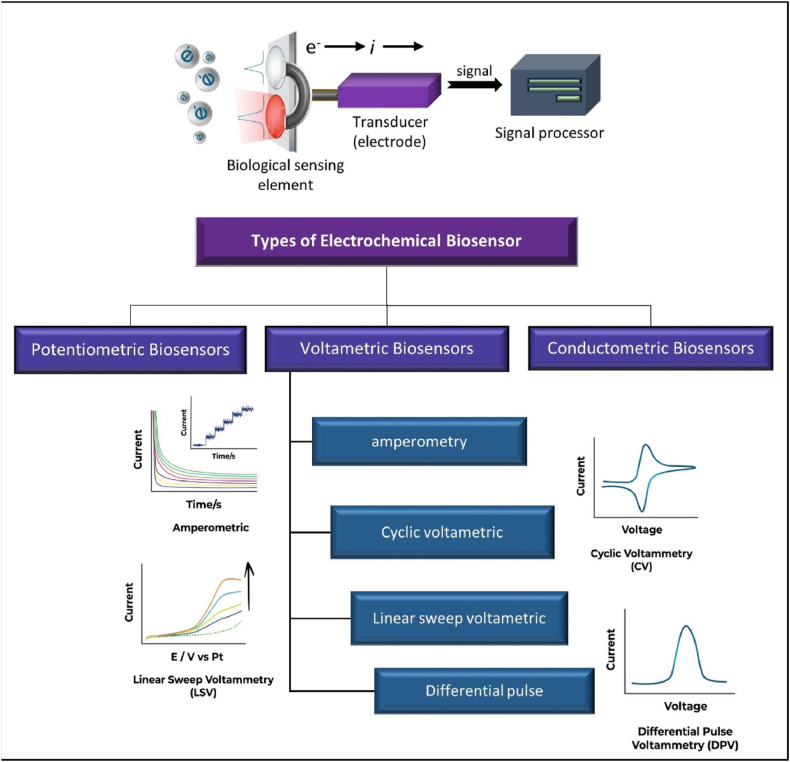
The types of electrochemical biosensors with the general mechanism.

Amperometric sensors measure redox reactions under constant potential, influenced by analyte concentration (Fig. 2). Enzymes are used due to their fast reaction velocity, high specificity, and immobilization feasibility. However, natural enzymes have proteinic disadvantages. Nanozymes, nanomaterials with enzymatic properties, have been developed to overcome these issues [26].

Liu et al. developed new amperometric biosensors based on tongue depressors and dental floss to detect saliva analytes. For the first time, they demonstrated the fabrication of an amperometric biosensor on a dental instrument. Glucose is utilized as a typical and frequent analyte for the sensor because it is also present in saliva. Hydrogen peroxide (H_2_O_2_) is an enzymatic product from glucose and is also found in saliva. Although these findings are promising, more research is needed to better understand the relationships between sensing performance and real-world oral conditions, in addition to optimizing the sensor fabrication technique and combining it with a PCB or chip for wireless communication [27].

Potentiometric biosensors are essential for estimating substances like glucose in clinical settings. They are also adaptable tools with potential applications in industry, security, environmental safety, and health. Recent improvements in potentiometric biosensors are meant to provide beginners with a general overview of the many types of potentiometric biosensors while also giving a concise but comprehensive evaluation of novel advances and trends for experienced practitioners. These trends include the utilization of nanomaterials, graphene, and novel immobilization materials, as well as a significant emphasis on tiny, flexible, and self-powered devices for in-field or at-home applications [28]. However, they face limitations such as interference, confounding variables, and application in sophisticated analytical scenarios. Potentiometric approaches need extensive knowledge of electrode properties and electrochemical concepts, which may limit their utility in clinical practice. Future advances in sensor design and cross-disciplinary cooperation can assist in overcoming these challenges and realizing the full potential of potentiometric methods for medical diagnosis and personalized medicine. Recognizing these restrictions and obstacles is crucial to widespread adoption and execution.

Conductometric biosensors offer a number of advantages. First, they do not require a reference electrode. Secondly, they operate at a low-amplitude alternating voltage, which prevents Faraday processes on electrodes. Thirdly, they are light-insensitive. Finally, they can be easily miniaturized and integrated using a low-cost thin-film technology. Berketa et al. studied the feasibility of adding an extra enzyme to a monoenzyme oxidase-based biosensor to improve its analytical properties. The developed approach has been effectively employed to optimize conductometric enzyme biosensors based on oxidases [29].

An electrode is a critical component that serves as a firm foundation for the immobilization of biomolecules (enzymes, antibodies, and nucleic acids) and electron transport. Furthermore, a biosensor consists of an electrochemical transducer and biorecognition elements (enzymes, antibodies, nucleic acids, etc.).

### 2.3 Mechanisms of electrochemical detection

Electrochemical sensing typically involves a reference electrode, a counter or auxiliary electrode, and a working electrode, also known as the sensing or redox electrode. The reference electrode, which is generally constructed of Ag/AgCl, is kept away from the reaction site to maintain a known and constant voltage. An electrochemical reaction requires the presence of both chemical species (neutral molecules or ions) and electrons. There are two types of reactions: anodic reactions, which release free electrons in the metal, and cathodic reactions, which accept those electrons.

## 3 The advancement of biosensors

### 3.1 Concept of biosensors

Biosensors are analytical instruments that use biological molecules to detect specific analytes or compounds. They include a biological recognition element, a transducer, and signal processing components. Biosensors have advantages such as high sensitivity, specificity, and fast response times, making them valuable in medical diagnostics, environmental monitoring, food safety, agriculture, bioprocessing, and the military. Recognition elements, transducer technologies, sensitivity, selectivity, downsizing, microfabrication, and data processing advancements are all aimed at improving detection limits, expanding the analyte range, increasing dependability, and enabling real-time monitoring [30].

### 3.2 Past, present, and future biosensors

**Fig. 3:**
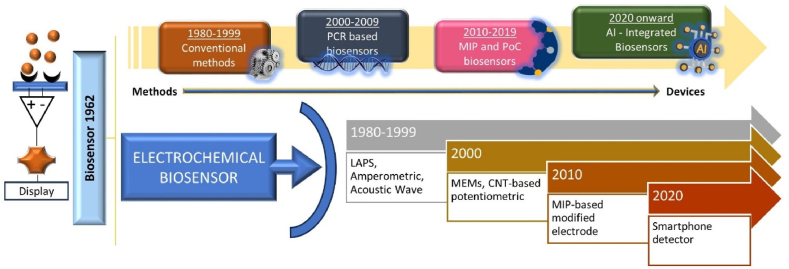
Paradigm shift in the field of development of electrochemical biosensors in advances.

Biosensors have the potential to revolutionize medical diagnostics [31,32], surpassing standard in vitro diagnostics for sickness detection and health monitoring. Scientific research is focused on improving their development. Fig. 3 illustrates the historical viewpoint and major discoveries in biosensor development for biological detection. In the 1980s, traditional diagnostic techniques were widely accepted as the gold standard for diagnosing infectious infections.

Chemometrics (the discipline of linking measurements taken on a chemical system to its state using mathematical or statistical techniques) is critical for biosensor detection, analysis, and diagnosis. In recent years, machine learning (ML) has made considerable progress in the field of artificial intelligence. However, the biosensor sector has lacked innovative and powerful machine learning approaches, particularly deep learning, which is widely used in facial recognition, image analysis, and speech recognition. This is despite AI capabilities and advances in disease control. The potential of smart biosensors to assist physicians in monitoring and forecasting illnesses for early intervention by sensing crucial variables is considerable. The artificial intelligence-powered biosensor gathers physiological and other data from patients' wearable biosensors and employs AI or machine learning algorithms to identify alterations in crucial signal patterns. Researchers are developing internet of medical things (IoMT)-assisted advanced biosensor technologies for point-of-care testing (POCT) of infectious diseases [17].

The combination of nanotechnology with smartphones has resulted in smart nanosensors, which could enable the common population to utilize cellphones as electrochemical, colorimetric, and fluorimetric sensors. Building a paper-based plasmonic biosensor that connects to a smartphone to automatically detect interleukin using augmented reality is a crucial step in COVID-19 diagnosis. Smartphones are used to detect fluorimetric and colorimetric changes, which are captured and measured using the smartphone camera and other android software. They may also detect electrochemical changes, which are subsequently analyzed with a smartphone camera and CMOS detectors to aid in data elaboration and transmission. According to some research groups, a novel all-fiber Fresnel reflection microfluidic biosensor (FRMB) was built by combining a microfluidic device and an all-fiber optical system [33,34].

The second-generation antibody-modified multimode fiber bioprobe can identify SARS-CoV-2 IgM and IgG antibodies to the spike protein in 7 min utilizing the Fresnel reflection approach [33]. The detection limits for SARS-CoV-2 IgM and IgG were 0.82 and 0.45 ng/mL, respectively. This simple approach has considerable potential for point-of-care diagnostics in combating COVID-19 and future pandemics. 88 Diagnosing pathogenic illnesses like COVID-19 and influenza requires careful consideration of spreading events, longer incubation durations, potential consequences, and available treatments. A recent study presents a label-free, self-powered, and ultrafast immunosensor device based on the triboelectric effect. To identify SARS-CoV-2 and H1N1, the equilibrium constants for particular antibody-antigen reactions are paired with antigen electric charges that are important to IEP. Optimizing simulation features, such as fluid flow and geometrical parameters, results in the best capture effectiveness of 86 percent [35].

### 3.3 Wearable technology in biosensors

Wearable technology in biosensors entails incorporating biosensing capabilities into wearable devices, enabling real-time monitoring of physiological parameters, biomarkers, or environmental conditions [13,36,37]. These devices provide ease, comfort, portability, and tailored health information. They can monitor physiological indicators such as heart rate, blood pressure, and activity levels, track biomarkers associated with health issues, and provide environmental monitoring to help make informed decisions [13]. They frequently integrate with mobile applications or cloud-based systems for data analysis, and they have simple user interfaces for visualization and feedback. They can also work with other devices to provide full health monitoring and management solutions. Wearable biosensors have the potential to completely transform healthcare, wellness, fitness, and environmental monitoring [38,39].

#### 3.3.1 Recent developments in AI-assisted wearable biosensors

Researchers are integrating AI-assisted wearable biosensors with smartphone-based sensing systems for the purpose of health and fitness monitoring, thereby creating a potential for the development of personalized healthcare and telemedicine systems. A differential cardiopulmonary monitoring system has been developed for the purpose of monitoring the health of patients infected with the SARS-CoV-2 virus, as well as workers and athletes [40]. The device employs inertial measurement units to measure the movement of the skin due to respiratory and cardiac activities. It is designed to integrate with a Bluetooth Low Energy (BLE) System on Chip, flash memory, lithium battery, and wireless charging unit [22]. The device's high accuracy in identifying coughing activities allows for improved patient care and pandemic management.

The wearable AI-biosensor networks (WAIBN), a clinical wearable biosensor network, consists of AI-assisted biosensors, personal devices, and cloud servers [20]. These biosensors collect pathological and physiological information, which is processed and analyzed by AI algorithms. The technology has potential in medical IoT applications, improving service quality and service costs. However, WAIBN needs higher energy efficiency, a longer lifetime, a lower delay, and reliability [22]. A cross-layer design optimal method was proposed to optimize these performance metrics, increasing data transmission reliability and lifetime. Amrita IoT Medical (AIM) Smart-edge is an IoT-based smart edge system developed using wearable biosensing devices [22]. Clinically validated, it demonstrated 87 % precision in remote medical monitoring, reduced energy costs and bandwidth, and increased network accessibility. Fog-empowered computation technology, used for anomaly detection, has shown potential in medical applications, reducing energy consumption and latency.

Qureshi et al. (2023) provide an in-depth analysis of the role machine learning (ML) plays in enhancing medical imaging, particularly in improving diagnostic accuracy. The research focuses on using ML in precision medicine, in which massive datasets are evaluated to enable individualized therapy based on specific patient profiles. Additionally, the paper discusses the integration of biosensors into the Internet of Things (IoT) ecosystem, showcasing their real-time monitoring capabilities for detecting physiological and chemical signals. This integration is made achievable by improvements in computer technologies, such as accelerated AI and edge computing, which allow for faster data processing and decision-making at the device level. The review outlines several challenges, including issues related to data quality, computational complexity, and the need for robust privacy and security measures. It also emphasizes the transformative potential of AI-powered biosensors in revolutionizing healthcare and patient care delivery [41]. Similarly, the works by Nittala et al. (2020, 2021) emphasize the use of ML for the design of compact physiological sensors with high-quality signal acquisition. Developing such sensors is a complex task that typically relies on heuristics and requires extensive expertise and training. Their research focuses on interactive textiles and ultrathin, skin-conformal patches that are intended to record signals like electrocardiograms (ECG), electrodermal activity (EDA), and surface electromyography (sEMG). These studies offer valuable design recommendations for improving the performance of each modality, informed by the latest advancements in AI and sensor technologies [42,43].

**Fig. 4:**
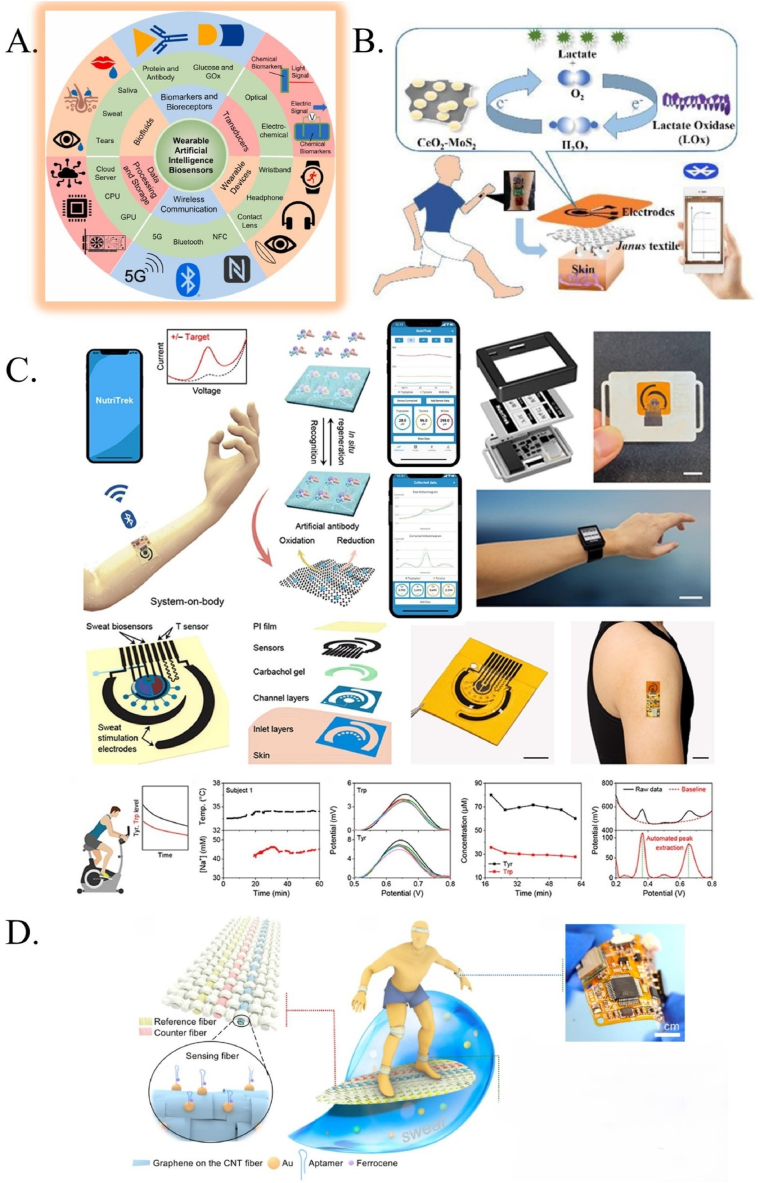
A. Functional components of AI-assisted wearable biosensing systems [46]. B. nanozyme–enzyme electrochemical biosensor for sweat lactate monitoring [47]. C. The ‘NutriTrek’ wearable biosensor for metabolic monitoring through sweat biosensing as a mobile application and a smartwatch and wearable system evaluation with custom voltammogram analysis using real-time calibrations [44]. D. A wearable electrochemical fabric for cytokine monitoring [48].

Recently, a wearable electrochemical biosensor has been developed to continuously analyze trace levels of metabolites and nutrients in sweat during physical exercise and at rest, as shown in Fig. 4B–D. The biosensor uses graphene electrodes, functionalized with metabolite-specific antibodies, and integrates with modules for sweat induction, sampling, signal processing, and wireless communication. In situ sweat analysis is difficult due to complicated sweat composition and requires technological advancements for precise on-body sensing (Fig. 4A). Direct LEG-MIP Trp sensing enables greater current peak heights after Tryptophan (Trp) recognition and binding into MIP cavities, resulting in more accurate and selective measurements [44].

Evaluation of a wearable system for dynamic physiological and nutritional monitoring [45]. The wearable technology was first evaluated by measuring sweat Trp and Tyr in human subjects during a bicycle exercise experiment with a constant load. The DPV data from the sensors was wirelessly transmitted to the mobile app, along with temperature and Na^+^ sensor readings, which automatically extracted the oxidation peaks using a custom-developed iterative baseline correction algorithm and calibrated for accurate sweat tyrosine (Tyr) and Trp quantification (Fig. 4B).

## 4 AI-application in electrochemical biosensor

### 4.1 Development of AI

Artificial intelligence, as one of the most significant scientific advances in the past few decades, has captivated the curiosity of academics from almost every field. In the 1990s, AI technologies became more widespread, and machine learning became more prevalent in traditional research. Recently, numerous AI approaches have been developed as affordable and effective substitutes for traditional mathematics and methods. They have also been proven to be particularly effective in dealing with the complicated nature of evolving, interactive, and uncertain circumstances [9,20].

AI-based environmental analysis is critical because of its capacity to rapidly evaluate massive amounts of data and detect subtle environmental changes. Machine learning (ML) is a branch of artificial intelligence that creates computer systems that learn from their experiences and adapt to new datasets. It is used to identify patterns, trends, and inefficiencies and make data-driven decisions. Learning can be supervised, unsupervised, semi-supervised, or reinforcement learning, and all of these methods use algorithms [49].

Supervised learning entails training a computer to recognize a certain pattern [50], such as detecting colors in photographs. The system employs a variety of mapping methods, including neural networks, support vector machines (SVMs), logistic regression, Bayesian classifiers, kernel machines, decision forests, and decision trees. In unsupervised learning, a computer is used to identify unknowns without the provision of feedback [50]. Semi-supervised learning is used to distinguish between supervised and unsupervised learning [51,52]. Reinforcement learning seeks to optimize reinforcement signals by mapping situations to actions, with training data indicating the appropriateness of the actions.

#### 4.1.1 Artificial neural networks (ANN)

**Fig. 5:**
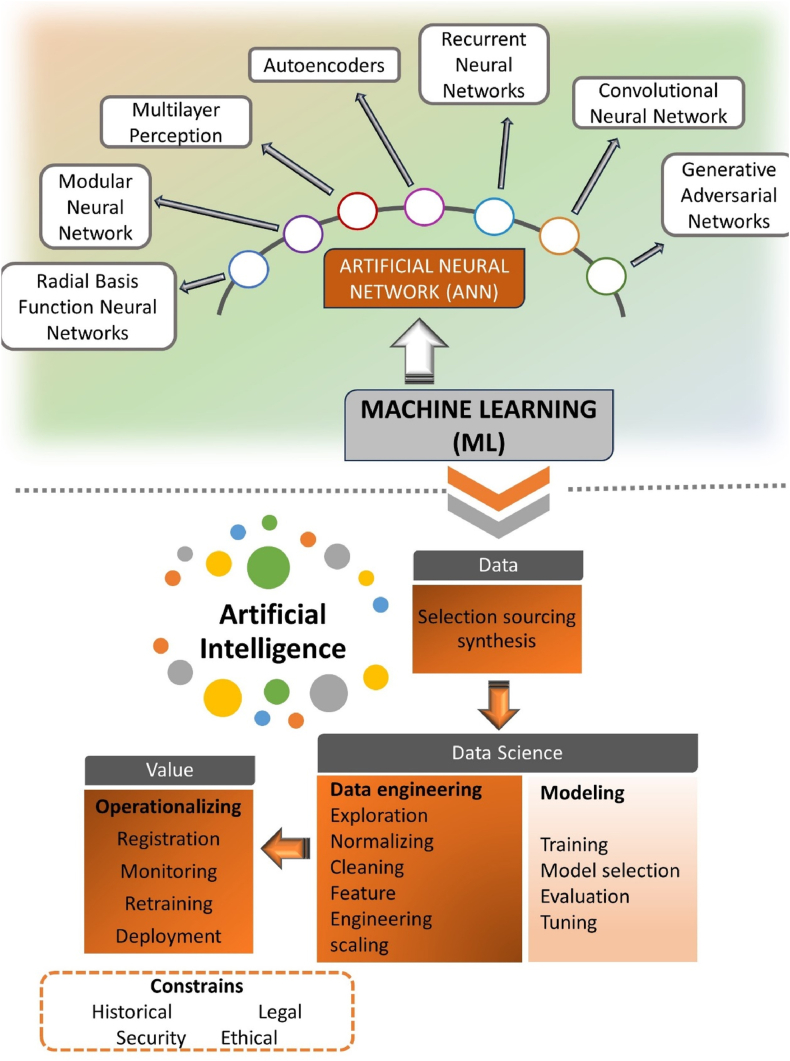
Machine learning algorithms and their connection to artificial intelligence data treatment.

Artificial neural networks (ANNs) are widely used machine learning tools for learning sophisticated data mining techniques and nonlinear statistical modeling (Fig. 5). These human nervous system-inspired algorithms are computer units that process input, generate output, and require little mathematical description, making them an attractive alternative to standard empirical modeling [9].

Artificial neural networks (ANN) are widely used techniques for analyzing data from multisensor arrays (MSA) and multisensor systems (MSS). However, adapting the system to changes during electrode exploitation and the variability of the medium or object under investigation is difficult. Intelligent data extraction and treatment approaches can help MSSs and MSAs perform better and be more practical. The pharmaceutical business is a promising field for developing smart MSAs and MSSs, with benefits including tailored pharmaceutical choices, doses, and increased communication between pharmacists and scientists. Recently, researchers used smart electrochemical MSS to forecast insulin dosage and glucose levels in diabetic patients. Traditional data treatment approaches failed because sensor responses overlapped. AI systems accurately extracted data and projected concentrations [12].

**Fig. 6:**
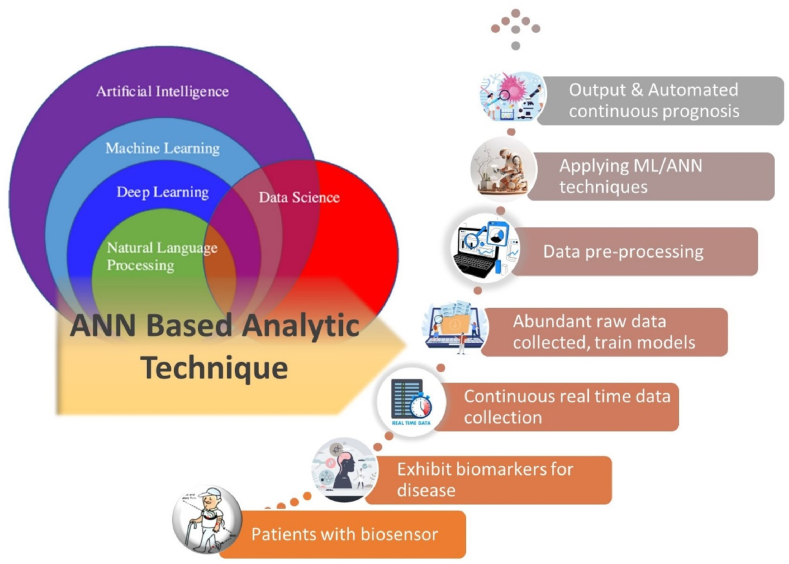
Relationship in AI component and Schematic of an ANN-based analytical technique.

To assess the effectiveness of ANN (ML) models in evaluating biosensor data for chronic diseases [53], we first define key terms and methodologies. This will assist us in gaining insight into the studies in these areas. The efficacy of artificial neural networks (ANN) is evaluated through the utilization of diverse machine learning (ML) methodologies and an array of ANN topologies. The advantages of these methodologies include the necessity for smaller data sets, which reduces the complexity of AI training. However, because drug and therapy data are typically fixed, models that are updated with fresh data must be evaluated on an ongoing basis to verify that the predictions remain accurate. This task presents a significant challenge. Although the terms “ANN” and “AI” are frequently used interchangeably, Fig. 6 depicts the link between AI, ML, deep learning (DL), natural language processing, and data science [54,55]. Fig. 4 also depicts an analytical technique using artificial neural networks for tailored health quality monitoring. In this concept, tailored and wearable biosensors detect biomarkers and measure data. This data is then preprocessed and analyzed by artificial neural networks in order to diagnose diseases and assess the likelihood of future disease progression [56].

Besides, Zeng and colleagues proposed that a unique LEEAS with dual photoelectric signal outputs could be a foundation for developing photoelectric hybrid artificial neural networks with high connectivity density and low latency [57]. Furthermore, photoelectric weight coordination improves the design flexibility and fault tolerance of artificial neural networks, paving the way for the next generation of optoelectronic, interconnected, brain-like computing systems.

**Fig. 7:**
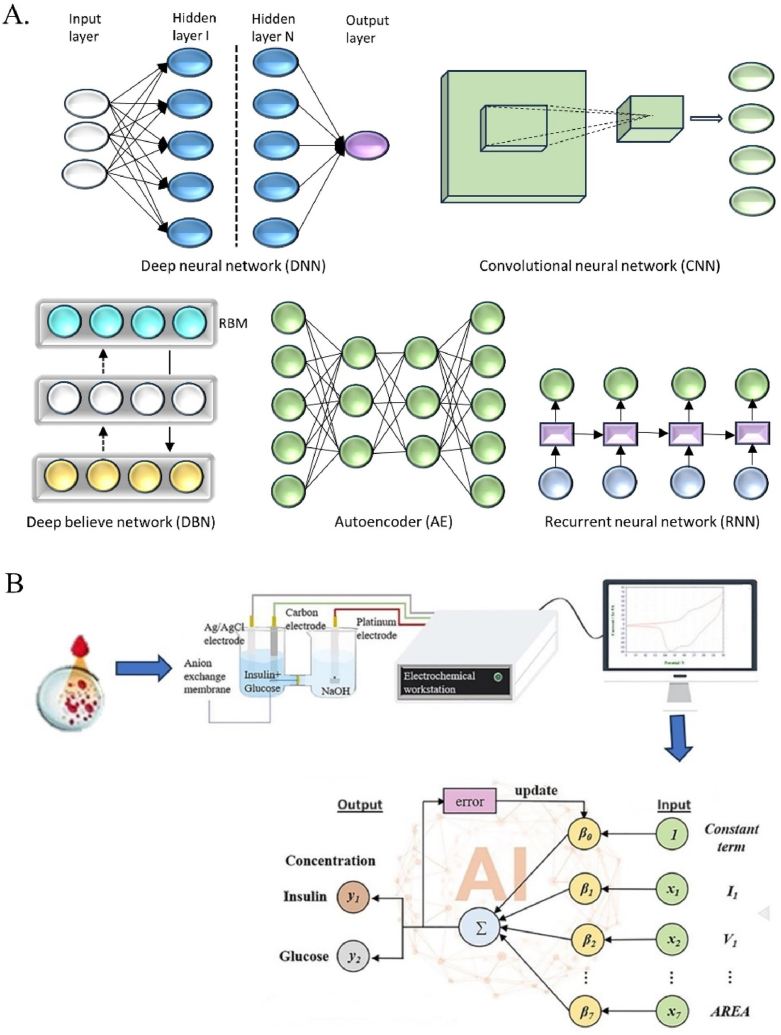
A. The structure of deep learning models. B. AI-coupled electrochemical experiment.

Deep learning is a machine-learning approach that enables the extraction of patterns and characteristics from large datasets. Complex neural networks comprising multiple hidden layers have been successfully trained. These neural networks are designated as “deep” due to the multitude of computational units comprising their numerous layers. Deep networks are capable of obtaining representations of information in exceedingly high-dimensional regions. A variety of deep learning models are currently available. In general, the models include convolutional neural networks (CNNs), deep belief networks (DBNs), auto-encoders (AEs), and recurrent neural networks (RNNs), as illustrated in Fig. 7A.

#### 4.1.2 Response surface methodology (RSM)

Statistical Design of Experiments (DoE) is an effective technique for optimizing chemical processes. Other techniques, as well as analysis of variance (ANOVA) and half-normal plots, can present this analysis. Contour plots and response surface methods can display data from nonlinear reaction investigations. Design Expert (Stat-Ease, Inc.), the latest DoE software program, enables users to create studies of varying complexity, including fractional factorial designs and response surface methodology (RSM) designs with multiple levels [58].

Response surface methodology (RSM) uses statistical techniques to investigate how two or more factors affect a particular response. It explains empirically how these variables interact with each other and have an impact on the response [59]. RSM commonly examines how multiple factors influence each other and their impact on the response. This technique effectively optimizes a wide range of processes, including nanocomposites [60].

According to Ashakirin et al., response surface methodology is utilized to maximize the impacts of potential and time intervals to boost sensitivity under ideal conditions. The RSM approach was used to optimize the potential value and time interval of Cu_2_O@MIP/SPCE, which affects the creatinine (Crn) oxidation current signal. The Cu_2_O@MIP sensor's sensitivity was tested using chronoamperometry to demonstrate the correlation between electrocatalytic oxidation current and Crn concentration. As a result, it improved the sensitivity and detection limits of the fabricated sensor [61].

Analyzing RSM optimization by a simultaneous determination of Cd (II) and Pb (II) using FeNi_3_/CuS/BiOCl was done previously. This research evaluation uses the CCD model to interact with the input variables. The three-dimensional surface plots between every two independent variables based on the equation show that pH, accumulation potential, accumulation time, and concentration of modifier have been significant factors in the electrochemical response of Pb (II) [62]. In addition, Design-Expert software includes mathematical statistics functions such as Box-Behnken Design (BBD), Central Composite Design (CCD), and Historical Data Design. The software allows users to create interactive 2D maps, place markers, and explore contours before seeing the response surface from all angles using a rotating 3D map. Ban et al. employed the Box-Behnken design to optimize the experiment's response surface. It demonstrated the practicality of employing the response surface approach to optimize experimental results as well as the efficacy of the dynamic diaphragm system electrochemical method for the degradation of ibuprofen [63]. Unlike RSM-based approaches, ANNs do not require a prior specification of a well-fitting function. Additionally, their universality in approximation allows them to approximate practically any type of nonlinear function, including quadratic functions [64]. Due to RSM's limitations in predicting nonlinear systems, such as biological ones, it is often combined with ANN-based models to build a strong RSM-ANN hybrid [65].

#### 4.1.3 Genetic algorithm (GA)

The Genetic Algorithm (GA) is an AI-based optimizer that can optimize any ANN output when combined with data from the ANN model. It is based on the “Survival of the Fittest” principle and uses genetic operators like mutation, selection, and crossover. Thus, GA is considered the most appropriate method to handle optimization problems in biology, such as the interaction between microalgae and bacteria [66].

### 4.2 Electrochemical platforms incorporated in detection techniques

Microfluidic platforms are used to identify, categorize, characterize, and predict data using artificial intelligence [45]. The development of microfluidic devices using machine learning and artificial intelligence holds promise for next-generation monitoring systems [67]. Also known as AI-coupled electrochemical experiments, as seen in Fig. 7B. However, challenges include scaling up these technologies to an industrial level and ensuring high-quality data for accurate analysis. Some companies have commercialized microfluidic technologies for environmental applications, including the detection of volatile organic compounds and CO_2_, as well as for environmental and food testing. We can potentially use AI-powered microfluidic platforms for environmental remediation and analysis [9].

**Fig. 8:**
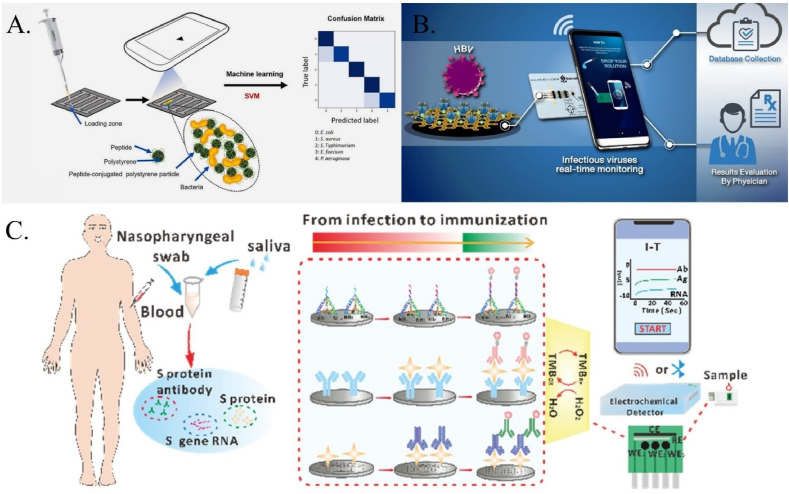
A. smartphone-based paper microfluidic chip used for the identification and classification of bacterial species [68]. B. Network-based drug repurposing for novel coronaviruses [69]. C. Universal biosensor linked smartphone for co-detection of SARS-CoV-2 viral RNA, antigen, and antibody [70].

Human sensor-inspired supervised machine learning for bacterial species classification using smartphone-based paper microfluidics (Fig. 8A). The study classifies bacterial species using a technique similar to the human tongue, with a small number of peptides acting as less-specific receptors. Supervised machine learning is used to classify species without the necessity for particular bioreceptors. The approach is portable, uses only four channels, and is unaffected by ambient illumination conditions. The mixed solution was then placed into the loading zone of the paper microfluidic chip, allowing it to flow through each channel spontaneously. Each chip includes four channels, and four distinct flows were monitored simultaneously by taking videos on a smartphone. A series of flow velocity values are evaluated from the video frames and used for the supervised machine learning model. In this case, the support vector machine (SVM) [68].

Using a Near Field Communication (NFC)-enabled smartphone, a label-free electrochemical immunosensor detects HBV by immobilizing antibodies with β-cyclodextrin (β-CD). A card-sized electrochemical NFC sensor was used for quantitative analysis (Fig. 8B). The amperometric detection procedure was managed by a smartphone running a proprietary, simple application. As an example of the method's usability, the described smartphone-based sensing device has the potential to be the next generation of portable and versatile POC tools for label-free electrochemical immunoassays. This electrochemical immunosensor has the potential to provide an alternative platform for portable, simple, sensitive, and selective tools that can easily assess a number of health markers using a low-cost sensor system controlled by a smartphone [69].

The development of a universal and portable three-in-one biosensor connected to a smartphone for the simultaneous detection of SARS-CoV-2 viral RNA, antigen, and antibody (Fig. 7C). Using a smartphone, it is possible to monitor SARS-CoV-2 virus-infected individuals from infection to immunization intelligently. Dou et al. describe a universal and portable three-in-one biosensor coupled to a smartphone to simultaneously detect SARS-CoV-2 viral RNA, antigen, and antibody [70].

#### 4.2.1 Electrochemical detection methods

Electrochemical techniques are an attractive option for detection methods due to their compatibility with miniaturization and integration with microfluidic devices. The efficient separation and detection of electroactive species in the presence of an electric field is a key advantage of electrochemical methods. The main advantage of electrochemical systems is their ability to integrate electrical parts without sacrificing performance due to miniaturization. Electrochemical detection techniques are an attractive option due to their potential for high selectivity (achieved through alterations to the electrode surface and characteristics), semi-selectivity (enabled by amperometric detection through potential manipulation), and universal applicability (enabled by conductivity detection). Recently, there have been considerable efforts to integrate electrochemical transducers with AI [71].

### 4.3 Artificial intelligence application

Artificial intelligence (AI) applications in electrochemical biosensors provide several advantages and breakthroughs in many areas of biosensing. Artificial intelligence is rapidly being applied to electrochemical biosensors to improve sensitivity, selectivity, reliability, and usability [65]. AI algorithms evaluate complicated signals, preprocess them, filter out noise, improve signal-to-noise ratios, and provide calibration and calibration-free sensing. AI also enables real-time monitoring, feedback control, and the integration of many sensing modalities [72].

A wearable electrochemical biosensor was created to allow real-time glucose monitoring for diabetes management [73]. The sensor generates an electrochemical signal proportional to glucose concentration by using a glucose oxidase enzyme [74]. The biosensor detects glucose levels in the interstitial fluid and sends the results wirelessly to a linked device. AI algorithms, such as machine learning models, analyze the data and predict blood glucose levels. The biosensor delivers real-time feedback to users, allowing for timely interventions. The AI models continuously learn and adapt to individual variances in glucose metabolism, sensor drift, and ambient conditions, thereby enhancing the accuracy and reliability of glucose monitoring [75]. This technique can be expanded to include other biomarkers and physiological characteristics, enhancing wearable biosensing technologies for a variety of healthcare applications.

The introduction of AI biosensors creates an opportunity for rapid access to wearable biosensors, thanks to the development of integrated commercial wearable sensors and the seamless integration of AI, big data, and IoT. In the future, there will be a greater interest in having quick access to wearable biosensors, which can be accomplished by merging sensors into WAIBN (Fig. 8) to capture a complete picture of the human subject [46,76]. Materials innovation, biorecognition elements, signal gathering, and data processing are among the challenges faced in this discipline.

**Fig. 9:**
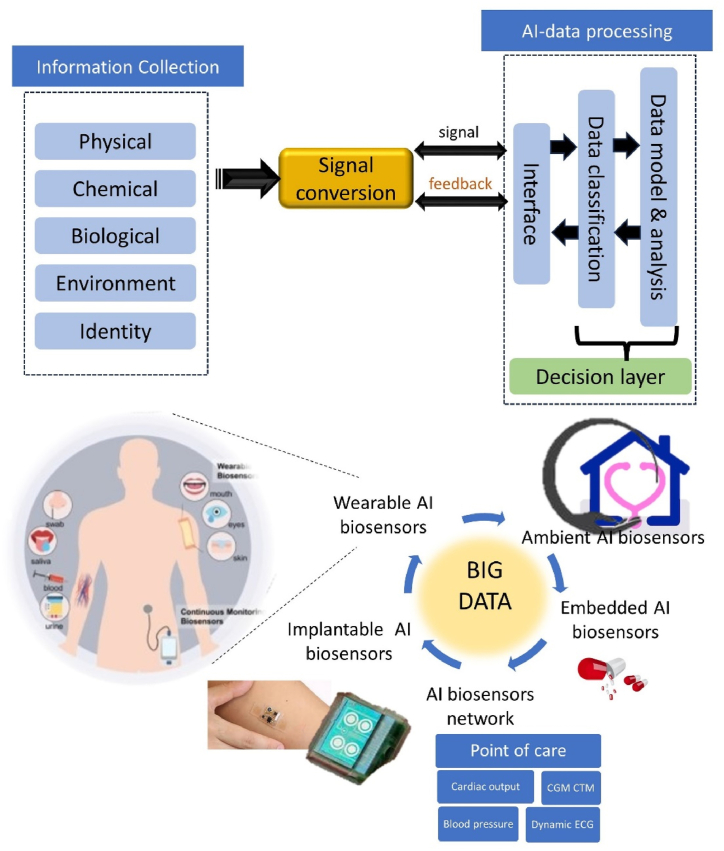
Application of AI-biosensor networks (AIBN).

AI-powered biosensors are being created for real-time monitoring in a variety of fields, including healthcare, assisted living, environmental monitoring, the military, biosafety, and more [77,78]. These networks can detect diseases early on, help people live longer lives, and improve their quality of life. They can also monitor the elderly's health and communicate during an emergency. AIBN applications can expand thanks to smartphones and smart cities (Fig. 9). As shown in Fig. 9, examples of AIBN include wearable, implantable, embedded, and ambient AI-biosensor networks. The interface can provide a user with information via a wireless data connection between AI-biosensors and smartphone-based platforms or other intelligent devices [59]. Big data processing in clouds can be pooled as needed.

### 4.4 AI biological detection using biosensors data

Here, we explore the use of AI and ML to analyze biomarker data for disease detection, including classification, regression, and clustering. Table 1 outlines the performance of machine learning approaches on various biomarker and symptom data from specific diseases.

**Table 1:** Performance of machine learning models for disease detection using biomarker and clinical outcomes (2018–2023).

Machine Learning	Studies	Outcome	Ref.
Deep learning system	Diabetic & cancer	Specificity: <96 %, sensitivity: <87 %	[82]
ANN	Lung cancer	Specificity: 96 %, sensitivity: 95.8 %	[83]
Naïve bayes and RF classifier	Blood cancer	Accuracy: 96.6 %	[84]
ANN	Breast cancer	Accuracy: 90 %	[85]
Gradient boosting	COPD	Accuracy: 91.3 %, sensitivity: 100 %	[86]
Gaussian Naïve Bayes classifier and SVM	Stroke	R2: 0.97	[87]
Decision support system	Dementia	AUC for DSI: 0.79; 0.75	[37]
-Designed unsupervised learning	Dementia	P-value: 0.024; 0.018	[13]
SVM classifier	CVD	Accuracy: 90.2 %	[75]
Feature extraction	oHCM	Accuracy: 99 %	[39]
ANN	Cardio.	Accuracy:>98 %	[88]
SVM and CNNs	Liver	–	[89]
Mann-Whitney-RF technique	Liver toxicity	FI: 0.91	[90]
Network-based prediction	COVID-19	–	[81]

Because of the pandemic COVID-19, an integrative antiviral drug repurposing technique has been developed that integrates a pharmacology-based network medicine system to quantify the interaction of the virus-host interactome and therapeutic targets in the human PPI network ANN [79]. These experiments are based on the assumption that proteins involved in viral infection (including HCoV) are located in the corresponding subnetwork of the comprehensive human protein-protein interaction (PPI) network. Additionally, proteins that act as potential therapeutic targets for a specific disease may also be suitable drug targets for antiviral infection due to the shared PPIs and functional pathways elucidated by the human interactome [80]. Researchers employ bioinformatics to validate drug-induced gene profiles and HCoV-induced transcriptomics in human cell lines to investigate the proposed mechanism of action of a specific HCoV for which they propose repurposing [81].

### 4.5 Discussion and lessons learned

According to the literature, machine learning provides enormous benefits to electrochemical processes. However, alternate methodologies and algorithms could prove more appropriate for specific uses. Furthermore, many new potential electrochemical applications of machine learning are largely untapped. Here, we also discuss each of the important areas of the literature, focusing on the future possibilities of machine learning research in electrochemical systems.

A perspective on the significance of machine learning in the electrochemical field has sparked increased interest and investigation. Mansour et al. conducted a comparative review of machine learning models in the electrochemical field. This paper discusses the latest glucose monitoring technologies, including invasive, minimally invasive, and non-invasive methods. It highlights the limitations and challenges of current solutions and suggests the integration of continuous glucose monitors and AI techniques for precise diabetes management protocols. It calls for further advancements in glucose monitoring technology. Another critical issue that has been addressed more thoroughly is electrochemical sensors' biofouling, particularly ISF-based ones [91]. Plank et al. emphasize machine learning's disruptive potential in the electrochemical domain. Their review of the distribution of relaxation times (DRT) analysis enhances the spectrum resolution and separability of electrochemical processes by decomposing frequency domain data into a distribution function of gains at relaxation times. It is useful for the characterization of materials and systems, and it may be used on spectra comprising electromagnetic effects, resistive-capacitive processes, and solid-state diffusion. A step-by-step methodology is described, and deconvolution is performed using probabilistic methods such as Tikhonov regularization. This method uses a Bayesian interpretation, which is well-known in classical machine learning [92]. Furthermore, Zhang et al.'s research demonstrates the competence of machine learning in the hierarchical modeling of electrochemical optimization and deterioration. A population-wide model can be developed using machine learning on a huge dataset and then modified with more customized aging and degradation models. This hierarchical strategy improves forecast accuracy while also allowing for a more detailed knowledge of electrochemical performance at community and individual levels [93]. In a nutshell, the research demonstrates that machine learning can dramatically boost productivity in the electrochemical domain by enhancing accuracy, resource consumption, and field data use.

Perspectives on the machine learning techniques used in various electrochemical system applications [[94], [95], [96]]. The literature contains several examples of increasing consensus regarding the usefulness of various machine learning methods for distinct electrochemical applications. Based on the literature, the following basic observations about particular tasks for each of the machine learning algorithms mentioned are proposed (Fig. 7A). As general insights into data quality and quantity, high-quality, huge datasets improve the performance of complicated models such as neural networks, although simpler models may do well with smaller datasets. The availability of computer resources influences algorithm selection, with deep learning models requiring more computing power and longer training times [97,98]. In some applications, interpretable models (e.g., decision trees, linear regression) are favored for scientific insights, while high-accuracy models (e.g., deep learning) are prioritized for performance optimization. Drawing on these collective views, researchers may make informed decisions on which machine learning algorithms to apply to specific electrochemical applications, resulting in improved system performance and new discoveries in the field.

Addressing these outstanding difficulties and capitalizing on highlighted opportunities has a chance to propel electrochemical machine learning research into an exciting future in terms of the value it adds to the larger electrochemical systems discipline. Indeed, one could claim that the most significant gaps and possibilities in electrochemical machine learning are for the study of new difficulties rather than the development of new solutions to previously explored problems.

## 5 Conclusion

Finally, the growing demand for an advanced engineering tool that can effectively manage the vast datasets generated by biosensors and health diagnostics is critical for the shift from traditional lab-based diagnostics to personalized healthcare devices. This review explores the integration of artificial intelligence (AI) with electrochemical biosensors for disease prediction and diagnosis, leveraging machine learning (ML) techniques. As for human subject application, biosensors evaluate biofluids for biomarkers indicating illness presence and severity. However, a single biomarker reading is often insufficient, necessitating the evaluation of multiple biomarkers over extended periods. The volume of data generated by biosensors is typically too large for manual analysis or basic mathematical methods, making ML algorithms particularly suitable for this task. These algorithms, especially artificial neural networks (ANNs), excel in processing and analyzing complex data, mimicking the pattern recognition capabilities of the human brain. Future research should focus on enhancing the accuracy and scalability of these AI-driven systems, enabling more precise, real-time disease monitoring, and fostering the development of truly personalized healthcare solutions. This study underscores the transformative potential of AI in advancing medical diagnostics and personalized treatment.

## 6 Challenges and future perspective

Biosensor fabrication presents hurdles since they must be small, customizable, wearable, and portable. Skin-based electrochemical sensors have advantages for detecting biomarkers and physiological indicators. However, nanomaterial-based biosensors must be functionalized in order to increase sensing performance, biocompatibility, low power consumption, and usability [94]. Another challenge is detecting several biomarkers from biofluids while avoiding interference from components such as saliva [95,96], perspiration, proteins [97], urea, and minerals [98]. Addressing these issues will require advancements in filtering technologies, as well as secure methods for biomarker data transmission and storage.

Another key aspect is the design and selection of machine learning algorithms, which are critical for increasing learning outcomes and data analysis in biosensor applications. The success of these algorithms depends on the availability of clean, abundant, and diverse datasets. Initial preprocessing and optimization of hyperparameters are essential for precise data mapping, especially for deep learning models that require ideal parameters for accurate prediction. ANN can generate valuable insights for personalized healthcare, but high-performance models demand rigorous training and evaluation frameworks.

Furthermore, AI applications can utilize early-stage chronic disease data to prevent or mitigate life-threatening symptoms by identifying critical biomarkers and correlating them with risk factors [50,99]. Despite these advances, challenges with data quality, AI model interpretability, and the incorporation of ethical considerations—such as data privacy, security, and algorithmic bias—must be addressed. The future direction of research should focus on refining AI-driven biosensor systems to enhance their accuracy, scalability, and reliability in healthcare, with an emphasis on real-time monitoring, personalized treatment plans, and ethical deployment in clinical settings. [100].

## CRediT authorship contribution statement

**Siti Nur Ashakirin Binti Mohd Nashruddin:** Writing – original draft, Visualization, Validation, Software, Resources, Investigation, Conceptualization. **Faridah Hani Mohamed Salleh:** Writing – review & editing, Supervision. **Rozan Mohamad Yunus:** Writing – review & editing. **Halimah Badioze Zaman:** Writing – review & editing.

## Declaration of competing interest

The authors declare that they have no known competing financial interests or personal relationships that could have appeared to influence the work reported in this paper.
